# Bis{(*S*)-(−)-*N*-[(2-biphen­yl)methyl­idene]-1-(4-meth­oxy­phen­yl)ethyl­amine-κ*N*}di­chlorido­palladium(II)

**DOI:** 10.1107/S2414314624005558

**Published:** 2024-06-16

**Authors:** Bertin Anzaldo, René Gutiérrez Pérez, Guadalupe Hernández Téllez, Ángel Mendoza, Gloria E. Moreno Morales

**Affiliations:** a Lab. Síntesis de Complejos, Fac. Cs. Quím.-BUAP, Ciudad Universitaria, PO Box, 72592 Puebla, Mexico; University of Antofagasta, Chile

**Keywords:** crystal structure, Schiff base, palladium(II) complex, monodentate

## Abstract

The Pd^II^ complex bis­{(*S*)-(−)-*N*-[(biphenyl-2-yl)methyl­idene]1-(4-meth­oxy­phen­yl)ethanamine-κ*N*}di­chlorido­palladium(II), [PdCl_2_(C_22_H_21_NO)_2_], crystallizes in the monoclinic Sohncke space group *P*2_1_ with a single mol­ecule in the asymmetric unit. The coordination environment around the palladium is slightly distorted square planar. The N—Pd—Cl bond angles are 91.85 (19), 88.10 (17), 89.96 (18), and 90.0 (2)°, while the Pd—Cl and Pd—N bond lengths are 2.310 (2) and 2.315 (2) Å and 2.015 (2) and 2.022 (6) Å, respectively. The crystal structure features inter­molecular N—H⋯Cl and intramolecular C—H⋯Pd inter­actions, which lead to the formation of a supramolecular framework structure.

## Abstract 2

The Pd atom is coordinated by two nitro­gen atoms from two *trans*-aligned imine ligands and two chlorine atoms in an essentially square-planar environment.

## Graphical abstract

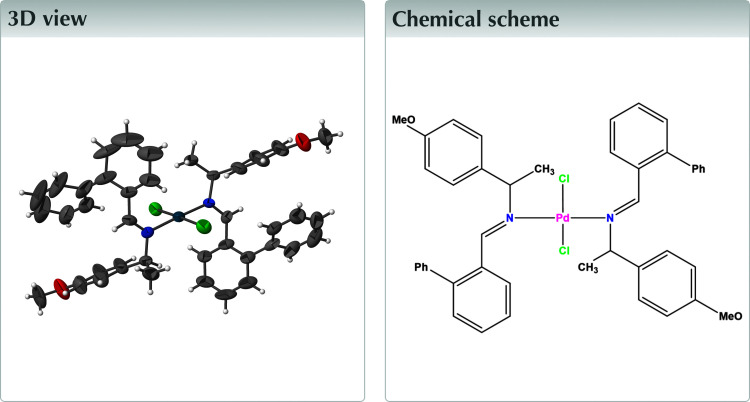

## Structure description

Schiff bases, derived from the condensation of primary amines and aldehydes, are well established and versatile ligands in coordination chemistry. Their flexibility has led to a diverse range of coordination complexes (Boulechfar *et al.*, 2023 ▸). Metal complexes with Schiff base ligands play crucial roles in enhancing catalytic efficiency in various chemical reactions, including oxidation, hy­droxy­lation, aldol condensation, and epoxidation (Gupta & Sutar, 2008 ▸; Brayton *et al.*, 2009 ▸; Bowes *et al.*, 2011 ▸). In addition to their catalytic capabilities, palladium(II) imine complexes exhibit significant biological potential. Their reactivity, influenced by electronic and steric factors, is highly tunable through substituent modifications, particularly with the introduction of chirality. Herein, we report the crystal structure of a novel palladium(II) complex [PdCl_2_(C_22_H_21_NO)_2_].

**Figure 1:**
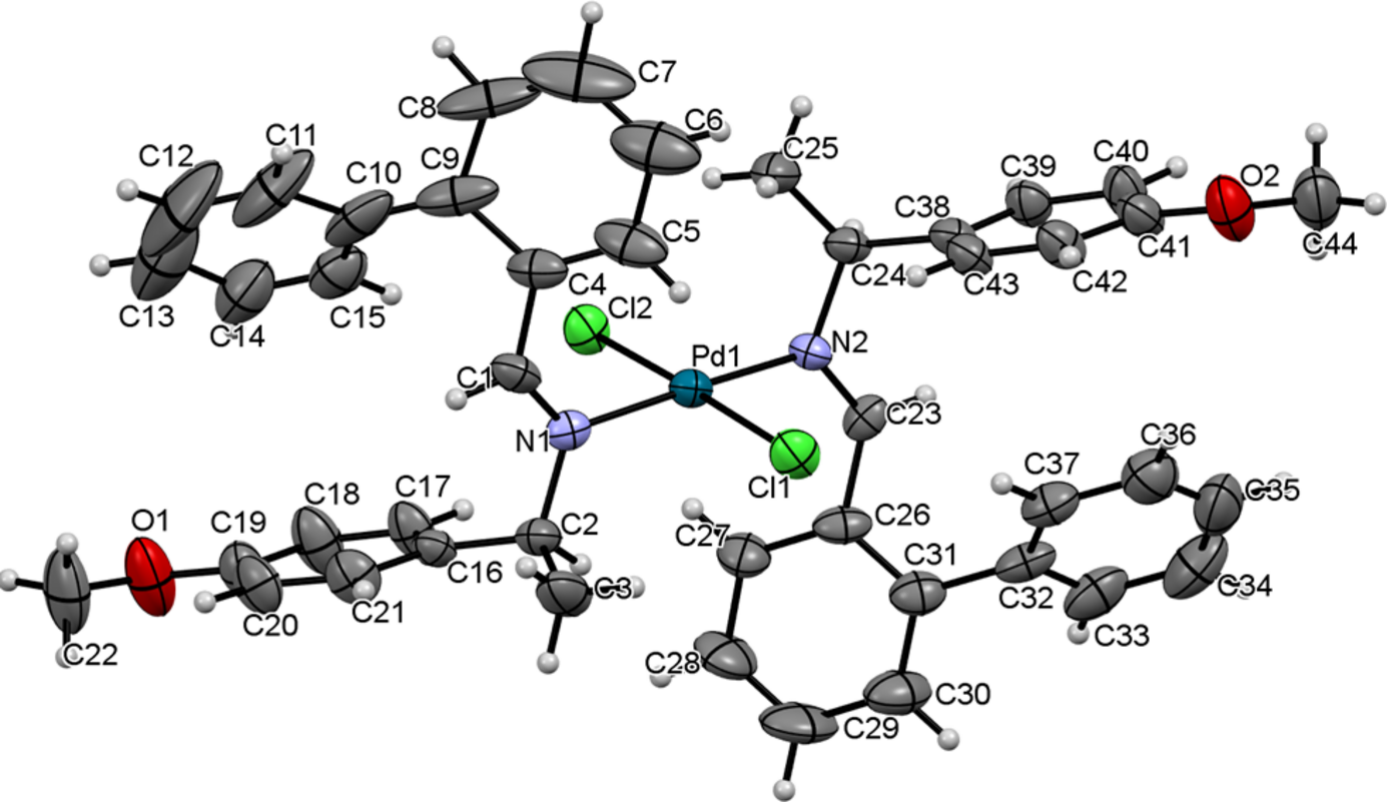
Mol­ecular structure of [PdCl_2_(C_22_H_21_NO)_2_]. Displacement ellipsoids are drawn at the 40% probability level.

The title Pd^II^ complex crystallizes in the monoclinic system with the *P*2_1_ space group. The structure of the *trans* complex, which contains a single mol­ecule in the asymmetric unit, is shown in Fig. 1 ▸. Inspection of the molecular structure confirms the expected square-planar coordination environment around the central palladium(II) atom. The two imine ligands coordinated to the Pd^II^ atom through their nitro­gen atoms in a *trans* configuration, with Pd1—N1 and Pd1—N2 bond lengths of 2.015 (6) and 2.022 (6) Å, respectively. The Pd—Cl bond lengths [Pd1—Cl1 = 2.310 (2) Å and Pd1—Cl2 = 2.315 (2) Å] fall within the expected ranges for this type of complex, which confirms the nature of the bonds. There is a slight distortion from the ideal square-planar geometry, as revealed by a deviation of 0.054 Å of the Pd^II^ atom from the plane defined by atoms Cl2–N2–Cl1–N1. The steric effects in the Pd^II^ complex are evident in the torsion angles C26—C23—N2—C24 [−175.5 (7)°] and C2—N1—C1—C4 [175.4 (7)°]. The N1—Pd1—Cl1 [91.85 (19)°] and N1—Pd1—Cl2 [88.10 (17)°] bond angles also deviate slightly from 90°, demonstrating steric influence. The bond lengths of the imine group are N2=C23 = 1.299 (9) Å and N1=C1 = 1.238 (10) Å. The bond angles [C1—N1—Pd1 = 124.5 (5)° and C23—N2—Pd1 = 122.7 (5)°] are slightly different. These bond lengths and angles, however, confirm the *sp^2^* hybridization of the C and N atoms.

The closest inter­molecular π–π stacking contact between the arene rings is 4.494 Å, which is above the typical range of 3.3–3.8 Å for favorable π–π inter­actions. Therefore, this inter­action does not significantly contribute to the cohesion of the crystal structure. The imine mean planes (C24—N2—C23 and C2—N1—C1) are twisted by 86 (2) and 85 (2)°, respectively, relative to the square-planar coordination mean plane (Cl2/Pd/Cl1). The two attached phenyl rings are not coplanar, as evidenced by the rotation of the mean plane C32–C37 with respect to the mean plane C26–C31 by an angle of 52.8 (4)°. Similarly, the mean plane C10–C15 is rotated with respect to the mean plane C4–C9 by an angle of 43.4 (6)°.

**Figure 2:**
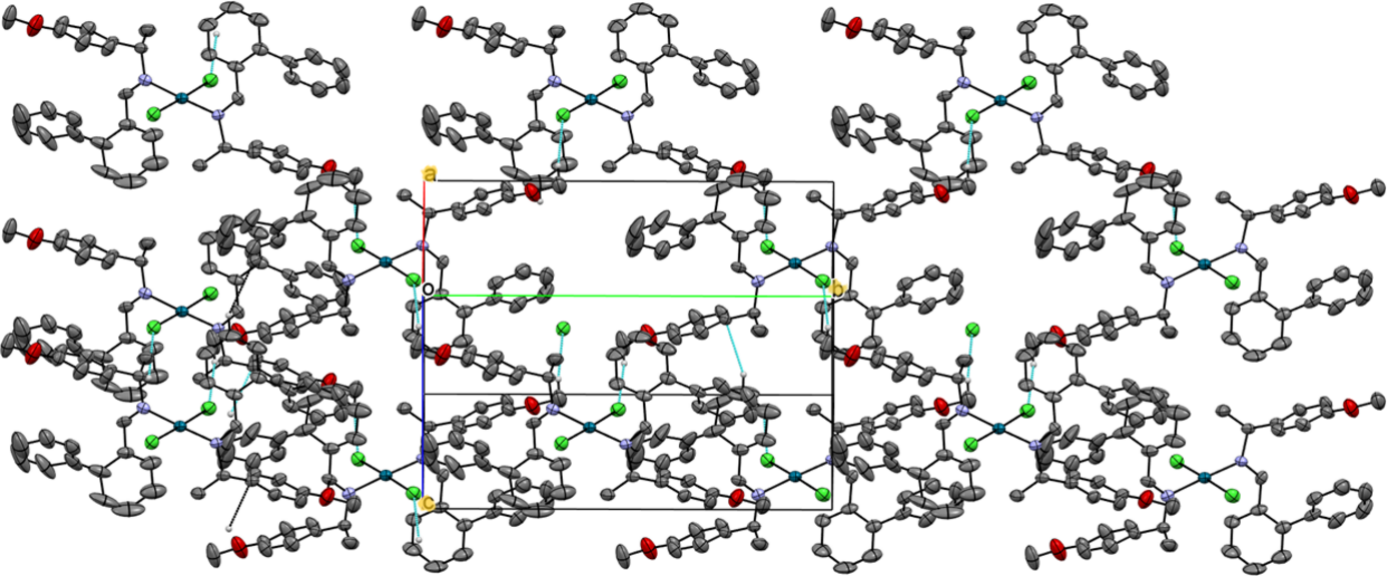
The crystal packing of the palladium(II) complex ialong [201]. The dashed lines indicate inter­molecular contacts. All H atoms not involved in these inter­actions have been omitted for clarity. Displacement ellipsoids are at the 40% probability level.

The complex mol­ecules are are stacked parallel to [001]. This arrangement is primarily driven by short-range van der Waals inter­actions and inter­molecular hydrogen bonds, particularly C—H⋯Cl inter­actions (Kinzhalov *et al.*, 2019 ▸), detailed in Table 1 ▸, which lead to a tri-periodic supramolecular framework (Fig. 2 ▸). The square-planar shape of the complex prevents the formation of Pd–Pd or π–π inter­molecular inter­actions, as evidenced by the shortest Pd⋯Pd distance of 10.634 Å and the shortest π–π distance of 4.494 Å, both exceeding van der Waals radii.

**Figure 3:**
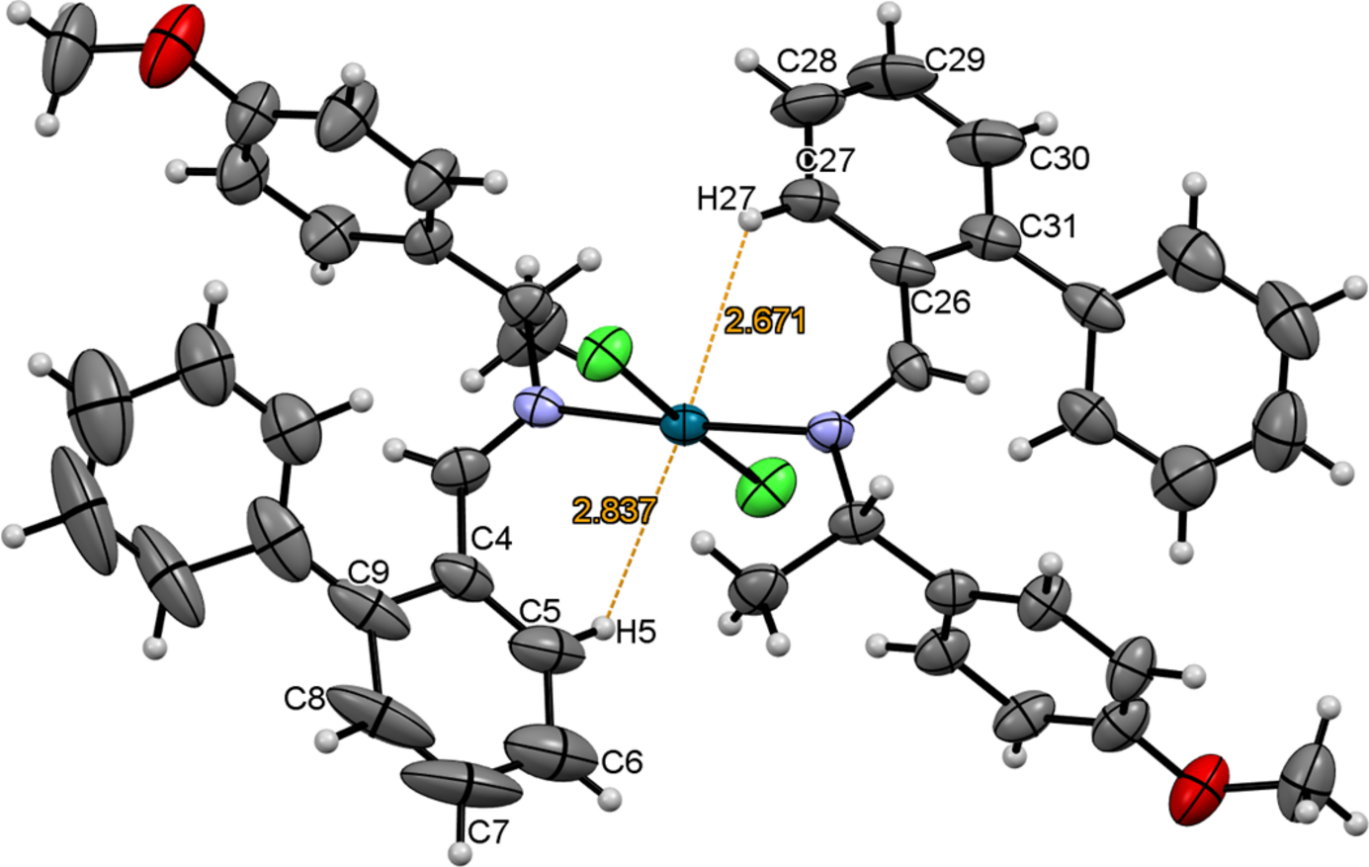
Pd⋯H inter­actions.

While the Pd⋯Pd distances exceed 10 Å, indicating minimal direct inter­action between palladium atoms, intra­molecular Pd⋯H inter­actions are observed (Fig. 3 ▸). These inter­actions are due to the specific orientations adopted by the phenyl rings (C26–C31 and C4–C9). The distances from the *ortho*-H atoms in these phenyl rings to the central Pd^II^ atom range from 2.67 Å (H27⋯Pd1) to 2.84 Å (H5⋯Pd1), suggesting a directional inter­action where the *ortho*-H atoms are oriented towards the Pd^II^ atom. These distances are shorter compared to the Pd⋯H distances involving the CH groups and CH_3_ groups within the complex.

A search of the Cambridge Structural Database (CSD, version 5.42, current as of February 2024; Groom *et al.*, 2016 ▸) revealed previously reported structures related to the Pd^II^ complex. UQUFIW (Duong *et al.*, 2011 ▸) crystallizes in space group *P*1. The chloride and (pyridin-4-yl)boronic acid ligands adopt a *trans* arrangement due to mol­ecular symmetry, with angles around 90°. FATQAU and FATPUN (Motswainyana *et al.*, 2012*b* ▸) crystallize in space group *P*2_1_/*n*. The two mol­ecular structures both exhibit a square-planar environment around the palladium atom. In each mol­ecule, the palladium(II) atom is coordinated by two *trans*-ferrocenyl­imine mol­ecules *via* their imine nitro­gen atoms, and either two chlorine atoms or a chlorine atom and a methyl group. The structure of LATNAV (Rochon *et al.*, 1993 ▸) exhibits hydrogen-bonding inter­actions between the hydroxyl groups and the chlorido ligands, with the Pd^II^ ion exhibiting a square-planar coordination environment around the central metal atom. YATQAN (Motswainyana *et al.*, 2012*a* ▸) in *P*2_1_/*n* exhibits a square-planar coordination environment around the palladium(II) atom, coordinated by two ferrocenyl­imine ligands *via* the imine nitro­gen atoms and chlorine atoms. The ferrocenyl­imine mol­ecules are *trans* to each other across the center of symmetry. The POCWEN (Anzaldo *et al.*, 2024 ▸) complex crystallizes in space group *P*2_1_, with the central atom tetra­coordinated by two nitro­gen atoms and two chlorine atoms, resulting in a square-planar configuration.

## Synthesis and crystallization

A solution of (*S*)-(−)-[1-(4-meth­oxy­phen­yl)-*N*-(2-biphen­yl)methyl­idene]ethyl­amine (0.100 g, 0.31 mmol) in di­chloro­methane (10 ml) was treated with bis­(benzo­nitrile)­palladium(II) chloride (0.060 g, 0.15 mmol) with stirring at room temperature for 8 h. After a few days, orange crystals of the title palladium(II) complex were obtained upon crystallization from a di­chloro­methane solution (yield 26%).

## Refinement

Crystal data, data collection and structure refinement details are summarized in Table 2 ▸.

## Supplementary Material

Crystal structure: contains datablock(s) I. DOI: 10.1107/S2414314624005558/bx4025sup1.cif

Structure factors: contains datablock(s) I. DOI: 10.1107/S2414314624005558/bx4025Isup2.hkl

CCDC references: 2333807, 2371278

Additional supporting information:  crystallographic information; 3D view; checkCIF report

## Figures and Tables

**Table 1:** Hydrogen-bond geometry (Å, °)

*D*—H⋯*A*	*D*—H	H⋯*A*	*D*⋯*A*	*D*—H⋯*A*
C3—H3*A*⋯Cl1	0.96	2.90	3.662 (6)	138
C22—H22*A*⋯Cl1^i^	0.96	2.87	3.765 (10)	155
C25—H25*A*⋯Cl2	0.96	2.71	3.460 (6)	135
C44—H44*C*⋯Cl2^ii^	0.96	2.82	3.757 (9)	165

Symmetry codes: (i) 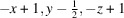; (ii) 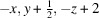.

**Table 2:** Experimental details

Crystal data	
Chemical formula	[PdCl_2_(C_22_H_21_NO)_2_]
*M*_r_	808.09
Crystal system, space group	Monoclinic, *P*2_1_
Temperature (K)	293
*a*, *b*, *c* (Å)	10.2505 (4), 18.6165 (9), 10.6345 (5)
β (°)	96.388 (4)
*V* (Å^3^)	2016.77 (16)
*Z*	2
Radiation type	Mo *K*α
μ (mm^−1^)	0.63
Crystal size (mm)	0.27 × 0.15 × 0.09

Data collection	
Diffractometer	Xcalibur, Atlas, Gemini
Absorption correction	Analytical *CrysAlis PRO* (Agilent, 2013 ▸)
*T*_min_, *T*_max_	0.938, 0.976
No. of measured, independent and observed [*I* > 2σ(*I*)] reflections	25419, 10009, 6673
*R*_int_	0.040
(sin θ/λ)_max_ (Å^−1^)	0.706

Refinement	
*R*[*F*^2^ > 2σ(*F*^2^)], *wR*(*F*^2^), *S*	0.041, 0.077, 1.02
No. of reflections	10009
No. of parameters	464
No. of restraints	108
H-atom treatment	H-atom parameters constrained
Δρ_max_, Δρ_min_ (e Å^−3^)	0.53, −0.30
Absolute structure	Flack *x* determined using 2435 quotients [(*I*^+^)−(*I*^−^)]/[(*I*^+^)+(*I*^−^)] (Parsons *et al.*, 2013 ▸)
Absolute structure parameter	0.00 (3)

Computer programs: *CrysAlis PRO* (Agilent, 2013 ▸), *olex2.solve* (Bourhis *et al* 2015 ▸), *SHELXL2019/2* (Sheldrick, 2015 ▸) and *OLEX2* (Dolomanov *et al.*, 2009 ▸).

## full crystallographic data

### (I) Crystal data

**Table:**

[PdCl_2_(C_22_H_21_NO)_2_]	*F*(000) = 832
*M**_r_* = 808.09	*D*_x_ = 1.331 Mg m^−^^3^
Monoclinic, *P*2_1_	Mo *K*α radiation, λ = 0.71073 Å
*a* = 10.2505 (4) Å	Cell parameters from 5972 reflections
*b* = 18.6165 (9) Å	θ = 3.4–25.6°
*c* = 10.6345 (5) Å	µ = 0.63 mm^−^^1^
β = 96.388 (4)°	*T* = 293 K
*V* = 2016.77 (16) Å^3^	Prism, clear gold
*Z* = 2	0.27 × 0.14 × 0.09 mm

### (I) Data collection

**Table:**

Xcalibur, Atlas, Gemini diffractometer	6673 reflections with *I* > 2σ(*I*)
Detector resolution: 10.5564 pixels mm^-1^	*R*_int_ = 0.040
ω scans	θ_max_ = 30.1°, θ_min_ = 2.9°
Absorption correction: analytical (CrysAlisPro; Agilent, 2013)	*h* = −14→14
*T*_min_ = 0.938, *T*_max_ = 0.976	*k* = −26→25
25419 measured reflections	*l* = −15→14
10009 independent reflections	

### (I) Refinement

**Table:**

Refinement on *F*^2^	Hydrogen site location: inferred from neighbouring sites
Least-squares matrix: full	H-atom parameters constrained
*R*[*F*^2^ > 2σ(*F*^2^)] = 0.041	*w* = 1/[σ^2^(*F*_o_^2^) + (0.0246*P*)^2^] where *P* = (*F*_o_^2^ + 2*F*_c_^2^)/3
*wR*(*F*^2^) = 0.077	(Δ/σ)_max_ < 0.001
*S* = 1.02	Δρ_max_ = 0.53 e Å^−^^3^
10009 reflections	Δρ_min_ = −0.30 e Å^−^^3^
464 parameters	Absolute structure: Flack x determined using 2435 quotients [(*I*^+^)-(*I*^-^)]/[(*I*^+^)+(*I*^-^)] (Parsons *et al.*, 2013)
108 restraints	Absolute structure parameter: 0.00 (3)
Primary atom site location: iterative	

### (I) Special details

**Table:**

Geometry. All esds (except the esd in the dihedral angle between two l.s. planes) are estimated using the full covariance matrix. The cell esds are taken into account individually in the estimation of esds in distances, angles and torsion angles; correlations between esds in cell parameters are only used when they are defined by crystal symmetry. An approximate (isotropic) treatment of cell esds is used for estimating esds involving l.s. planes.

### (I) Fractional atomic coordinates and isotropic or equivalent isotropic displacement parameters (Å^2^)

**Table:**

	*x*	*y*	*z*	*U*_iso_*/*U*_eq_	
Pd1	0.24859 (6)	0.40648 (10)	0.75111 (6)	0.03890 (9)	
Cl1	0.43721 (19)	0.47538 (12)	0.7684 (2)	0.0604 (6)	
Cl2	0.05859 (18)	0.33839 (10)	0.7241 (2)	0.0550 (6)	
O1	0.1719 (6)	0.0486 (4)	0.3702 (8)	0.100 (3)	
O2	0.3184 (6)	0.7648 (4)	1.1169 (7)	0.082 (2)	
N1	0.3499 (5)	0.3166 (3)	0.7207 (6)	0.0386 (16)	
N2	0.1404 (6)	0.4957 (3)	0.7708 (6)	0.0395 (16)	
C1	0.3795 (7)	0.2697 (5)	0.8014 (7)	0.052 (2)	
H1	0.416918	0.227848	0.773721	0.062*	
C2	0.3844 (7)	0.3062 (4)	0.5886 (7)	0.0483 (18)	
H2	0.336977	0.343456	0.536804	0.058*	
C3	0.5288 (6)	0.3208 (3)	0.5841 (6)	0.0785 (19)	
H3A	0.548930	0.368729	0.613494	0.118*	
H3B	0.549528	0.315922	0.498733	0.118*	
H3C	0.579616	0.287057	0.637410	0.118*	
C4	0.3616 (7)	0.2734 (6)	0.9367 (8)	0.060 (2)	
C5	0.3996 (7)	0.3345 (6)	1.0049 (8)	0.081 (2)	
H5	0.431888	0.373415	0.963132	0.097*	
C6	0.3909 (8)	0.3395 (7)	1.1334 (9)	0.115 (3)	
H6	0.414431	0.381245	1.178252	0.138*	
C7	0.3454 (11)	0.2792 (10)	1.1929 (10)	0.141 (5)	
H7	0.340691	0.280884	1.279651	0.169*	
C8	0.3086 (10)	0.2196 (9)	1.1305 (11)	0.124 (4)	
H8	0.276143	0.181801	1.175099	0.149*	
C9	0.3161 (11)	0.2106 (8)	0.9996 (11)	0.082 (3)	
C10	0.2782 (10)	0.1453 (7)	0.9353 (13)	0.089 (3)	
C11	0.3238 (11)	0.0769 (8)	0.9826 (14)	0.134 (5)	
H11	0.379967	0.074179	1.057397	0.160*	
C12	0.2858 (15)	0.0164 (8)	0.9193 (18)	0.169 (6)	
H12	0.320193	−0.027287	0.949817	0.203*	
C13	0.1997 (12)	0.0166 (8)	0.8132 (17)	0.152 (5)	
H13	0.170297	−0.026383	0.775614	0.182*	
C14	0.1561 (10)	0.0815 (6)	0.7616 (12)	0.102 (3)	
H14	0.101062	0.082413	0.685895	0.123*	
C15	0.1929 (10)	0.1431 (6)	0.8204 (11)	0.080 (3)	
H15	0.161244	0.186121	0.784604	0.096*	
C16	0.3373 (7)	0.2364 (4)	0.5340 (7)	0.0430 (18)	
C17	0.2051 (9)	0.2281 (6)	0.4933 (10)	0.061 (3)	
H17	0.148723	0.266325	0.503160	0.073*	
C18	0.1535 (9)	0.1657 (6)	0.4389 (11)	0.083 (3)	
H18	0.064439	0.162442	0.410954	0.100*	
C19	0.2347 (9)	0.1088 (5)	0.4264 (10)	0.062 (3)	
C20	0.3628 (9)	0.1128 (5)	0.4658 (9)	0.069 (3)	
H20	0.416800	0.073219	0.458349	0.083*	
C21	0.4150 (8)	0.1764 (5)	0.5179 (9)	0.063 (3)	
H21	0.504769	0.178980	0.542875	0.075*	
C22	0.2407 (10)	−0.0145 (6)	0.3697 (13)	0.112 (4)	
H22A	0.304857	−0.010854	0.310764	0.168*	
H22B	0.181398	−0.053147	0.345099	0.168*	
H22C	0.284092	−0.023545	0.452904	0.168*	
C23	0.1237 (6)	0.5452 (4)	0.6844 (7)	0.0441 (19)	
H23	0.085283	0.588047	0.706001	0.053*	
C24	0.0845 (7)	0.5131 (4)	0.8931 (6)	0.0453 (17)	
H24	−0.009558	0.521867	0.871482	0.054*	
C25	0.0971 (6)	0.4514 (3)	0.9839 (5)	0.0641 (15)	
H25A	0.052401	0.410297	0.945365	0.096*	
H25B	0.058729	0.464220	1.059108	0.096*	
H25C	0.188238	0.440139	1.005443	0.096*	
C26	0.1610 (7)	0.5390 (5)	0.5541 (7)	0.051 (2)	
C27	0.1426 (7)	0.4738 (5)	0.4885 (7)	0.0625 (19)	
H27	0.111914	0.433438	0.527477	0.075*	
C28	0.1712 (8)	0.4708 (5)	0.3644 (7)	0.085 (2)	
H28	0.158934	0.428004	0.319704	0.102*	
C29	0.2166 (8)	0.5289 (7)	0.3072 (8)	0.104 (3)	
H29	0.237239	0.525665	0.224424	0.125*	
C30	0.2324 (9)	0.5939 (6)	0.3724 (8)	0.084 (3)	
H30	0.262654	0.634092	0.332495	0.100*	
C31	0.2036 (9)	0.5990 (6)	0.4957 (9)	0.058 (2)	
C32	0.2216 (8)	0.6702 (5)	0.5639 (8)	0.056 (2)	
C33	0.1627 (9)	0.7300 (6)	0.5079 (9)	0.079 (3)	
H33	0.113274	0.725890	0.429438	0.095*	
C34	0.1758 (9)	0.7962 (6)	0.5664 (11)	0.097 (3)	
H34	0.138444	0.837068	0.527241	0.116*	
C35	0.2465 (9)	0.7998 (6)	0.6853 (12)	0.093 (3)	
H35	0.251564	0.843407	0.728319	0.111*	
C36	0.3078 (9)	0.7422 (6)	0.7400 (9)	0.082 (3)	
H36	0.358637	0.746573	0.817702	0.099*	
C37	0.2948 (8)	0.6752 (6)	0.6792 (9)	0.063 (2)	
H37	0.335477	0.634764	0.716872	0.075*	
C38	0.1443 (7)	0.5827 (4)	0.9508 (7)	0.0448 (19)	
C39	0.0664 (8)	0.6388 (5)	0.9723 (8)	0.054 (2)	
H39	−0.023526	0.635579	0.948671	0.065*	
C40	0.1192 (8)	0.7021 (5)	1.0298 (9)	0.062 (2)	
H40	0.064808	0.740340	1.045416	0.074*	
C41	0.2539 (9)	0.7064 (5)	1.0629 (9)	0.060 (3)	
C42	0.3310 (8)	0.6474 (5)	1.0453 (9)	0.056 (2)	
H42	0.420553	0.648662	1.071609	0.068*	
C43	0.2757 (8)	0.5872 (5)	0.9893 (9)	0.054 (2)	
H43	0.328990	0.548042	0.976853	0.065*	
C44	0.2485 (10)	0.8307 (6)	1.1136 (10)	0.091 (3)	
H44A	0.210510	0.840209	1.028576	0.136*	
H44B	0.307576	0.868873	1.141785	0.136*	
H44C	0.180116	0.827590	1.168128	0.136*	

### (I) Atomic displacement parameters (Å^2^)

**Table:**

	*U*^11^	*U*^22^	*U*^33^	*U*^12^	*U*^13^	*U*^23^
Pd1	0.03554 (14)	0.04656 (15)	0.03469 (15)	−0.00173 (13)	0.00435 (10)	−0.00068 (14)
Cl1	0.0433 (11)	0.0668 (16)	0.0728 (15)	−0.0116 (11)	0.0139 (10)	−0.0130 (13)
Cl2	0.0404 (10)	0.0581 (14)	0.0670 (14)	−0.0082 (10)	0.0081 (9)	−0.0118 (12)
O1	0.065 (4)	0.081 (5)	0.155 (7)	0.000 (4)	0.013 (4)	−0.050 (5)
O2	0.071 (4)	0.063 (5)	0.112 (5)	−0.008 (4)	0.015 (4)	−0.034 (4)
N1	0.030 (3)	0.048 (4)	0.036 (4)	−0.004 (3)	0.000 (2)	0.003 (3)
N2	0.041 (3)	0.046 (4)	0.032 (3)	0.001 (3)	0.007 (3)	−0.002 (3)
C1	0.048 (4)	0.065 (6)	0.045 (5)	0.002 (4)	0.017 (4)	−0.009 (4)
C2	0.055 (4)	0.052 (4)	0.040 (4)	−0.003 (3)	0.016 (3)	−0.001 (3)
C3	0.078 (4)	0.085 (4)	0.081 (4)	−0.029 (3)	0.044 (4)	−0.023 (4)
C4	0.049 (4)	0.088 (5)	0.045 (5)	0.022 (4)	0.013 (3)	0.017 (4)
C5	0.074 (5)	0.123 (6)	0.044 (4)	0.031 (4)	−0.002 (4)	−0.002 (4)
C6	0.093 (6)	0.194 (9)	0.056 (5)	0.043 (7)	0.000 (5)	−0.003 (5)
C7	0.104 (8)	0.278 (14)	0.040 (5)	0.022 (9)	0.002 (5)	0.021 (6)
C8	0.080 (6)	0.230 (12)	0.062 (6)	0.002 (7)	0.011 (5)	0.071 (6)
C9	0.047 (5)	0.142 (7)	0.059 (6)	0.016 (5)	0.013 (4)	0.046 (5)
C10	0.053 (6)	0.102 (7)	0.116 (8)	0.006 (6)	0.031 (5)	0.062 (6)
C11	0.075 (7)	0.127 (8)	0.199 (12)	0.008 (7)	0.018 (7)	0.112 (9)
C12	0.136 (12)	0.100 (7)	0.273 (17)	0.021 (8)	0.032 (10)	0.113 (10)
C13	0.123 (9)	0.089 (6)	0.249 (14)	−0.010 (6)	0.043 (9)	0.057 (8)
C14	0.098 (7)	0.076 (6)	0.138 (8)	−0.005 (5)	0.030 (5)	0.027 (5)
C15	0.066 (5)	0.081 (6)	0.096 (7)	−0.003 (5)	0.028 (5)	0.026 (5)
C16	0.042 (4)	0.048 (4)	0.041 (4)	0.003 (3)	0.013 (3)	−0.002 (3)
C17	0.040 (4)	0.062 (6)	0.081 (6)	0.010 (4)	0.007 (4)	−0.021 (5)
C18	0.053 (5)	0.081 (7)	0.115 (9)	0.003 (5)	0.008 (5)	−0.039 (6)
C19	0.054 (5)	0.053 (6)	0.080 (7)	−0.006 (4)	0.014 (4)	−0.015 (5)
C20	0.060 (5)	0.061 (6)	0.087 (7)	0.023 (4)	0.011 (5)	−0.012 (5)
C21	0.041 (4)	0.072 (7)	0.075 (6)	0.006 (4)	0.005 (4)	−0.006 (5)
C22	0.091 (7)	0.059 (7)	0.187 (12)	0.018 (6)	0.016 (7)	−0.034 (7)
C23	0.034 (4)	0.045 (5)	0.051 (5)	0.001 (3)	0.000 (3)	0.014 (4)
C24	0.043 (3)	0.059 (4)	0.035 (3)	−0.006 (3)	0.011 (3)	−0.003 (3)
C25	0.087 (4)	0.063 (4)	0.046 (3)	−0.014 (3)	0.021 (3)	−0.004 (3)
C26	0.043 (4)	0.080 (5)	0.030 (4)	−0.006 (3)	−0.002 (3)	0.008 (4)
C27	0.075 (5)	0.072 (5)	0.038 (4)	−0.003 (4)	−0.002 (3)	0.002 (3)
C28	0.115 (6)	0.103 (6)	0.037 (4)	−0.007 (6)	0.004 (4)	−0.017 (4)
C29	0.121 (8)	0.161 (9)	0.032 (4)	−0.024 (7)	0.015 (4)	−0.007 (5)
C30	0.093 (6)	0.116 (6)	0.042 (4)	−0.029 (5)	0.006 (4)	0.004 (4)
C31	0.053 (5)	0.076 (5)	0.044 (5)	−0.003 (4)	0.000 (4)	0.009 (4)
C32	0.043 (5)	0.075 (6)	0.052 (5)	−0.005 (4)	0.008 (4)	0.023 (4)
C33	0.064 (5)	0.085 (6)	0.088 (6)	−0.021 (5)	0.005 (5)	0.031 (5)
C34	0.076 (5)	0.080 (7)	0.132 (8)	−0.001 (5)	0.005 (5)	0.050 (6)
C35	0.101 (7)	0.059 (5)	0.124 (8)	−0.014 (5)	0.037 (6)	−0.002 (5)
C36	0.086 (5)	0.085 (7)	0.077 (6)	−0.015 (5)	0.021 (4)	0.001 (5)
C37	0.063 (5)	0.069 (6)	0.057 (5)	−0.001 (4)	0.008 (4)	0.017 (4)
C38	0.044 (4)	0.056 (5)	0.036 (4)	0.002 (3)	0.012 (3)	−0.001 (3)
C39	0.047 (4)	0.055 (5)	0.062 (5)	−0.003 (4)	0.015 (4)	−0.010 (4)
C40	0.056 (5)	0.051 (5)	0.083 (6)	0.002 (4)	0.023 (4)	−0.017 (5)
C41	0.060 (6)	0.064 (6)	0.061 (6)	−0.009 (5)	0.023 (4)	−0.018 (5)
C42	0.041 (4)	0.070 (6)	0.060 (5)	−0.009 (4)	0.009 (3)	−0.022 (4)
C43	0.054 (5)	0.060 (6)	0.049 (4)	0.004 (4)	0.013 (4)	−0.010 (4)
C44	0.095 (7)	0.061 (7)	0.122 (8)	−0.007 (6)	0.035 (6)	−0.018 (6)

### (I) Geometric parameters (Å, º)

**Table:**

Pd1—Cl1	2.310 (2)	C20—H20	0.9300
Pd1—Cl2	2.315 (2)	C20—C21	1.388 (12)
Pd1—N1	2.015 (6)	C21—H21	0.9300
Pd1—N2	2.022 (6)	C22—H22A	0.9600
O1—C19	1.394 (11)	C22—H22B	0.9600
O1—C22	1.370 (11)	C22—H22C	0.9600
O2—C41	1.364 (11)	C23—H23	0.9300
O2—C44	1.419 (11)	C23—C26	1.482 (10)
N1—C1	1.238 (10)	C24—H24	0.9800
N1—C2	1.500 (8)	C24—C25	1.497 (9)
N2—C23	1.299 (9)	C24—C38	1.533 (11)
N2—C24	1.512 (8)	C25—H25A	0.9600
C1—H1	0.9300	C25—H25B	0.9600
C1—C4	1.472 (11)	C25—H25C	0.9600
C2—H2	0.9800	C26—C27	1.402 (11)
C2—C3	1.511 (8)	C26—C31	1.373 (12)
C2—C16	1.482 (11)	C27—H27	0.9300
C3—H3A	0.9600	C27—C28	1.384 (10)
C3—H3B	0.9600	C28—H28	0.9300
C3—H3C	0.9600	C28—C29	1.349 (12)
C4—C5	1.382 (12)	C29—H29	0.9300
C4—C9	1.449 (15)	C29—C30	1.395 (13)
C5—H5	0.9300	C30—H30	0.9300
C5—C6	1.383 (12)	C30—C31	1.379 (12)
C6—H6	0.9300	C31—C32	1.512 (14)
C6—C7	1.393 (18)	C32—C33	1.370 (12)
C7—H7	0.9300	C32—C37	1.368 (12)
C7—C8	1.327 (19)	C33—H33	0.9300
C8—H8	0.9300	C33—C34	1.380 (14)
C8—C9	1.412 (16)	C34—H34	0.9300
C9—C10	1.427 (18)	C34—C35	1.387 (13)
C10—C11	1.429 (16)	C35—H35	0.9300
C10—C15	1.422 (15)	C35—C36	1.343 (13)
C11—H11	0.9300	C36—H36	0.9300
C11—C12	1.35 (2)	C36—C37	1.403 (12)
C12—H12	0.9300	C37—H37	0.9300
C12—C13	1.353 (19)	C38—C39	1.349 (11)
C13—H13	0.9300	C38—C43	1.366 (10)
C13—C14	1.380 (15)	C39—H39	0.9300
C14—H14	0.9300	C39—C40	1.408 (11)
C14—C15	1.340 (14)	C40—H40	0.9300
C15—H15	0.9300	C40—C41	1.389 (12)
C16—C17	1.385 (11)	C41—C42	1.378 (12)
C16—C21	1.394 (11)	C42—H42	0.9300
C17—H17	0.9300	C42—C43	1.362 (12)
C17—C18	1.377 (13)	C43—H43	0.9300
C18—H18	0.9300	C44—H44A	0.9600
C18—C19	1.362 (12)	C44—H44B	0.9600
C19—C20	1.336 (12)	C44—H44C	0.9600
			
Cl1—Pd1—Cl2	177.44 (11)	C20—C21—H21	118.9
N1—Pd1—Cl1	91.85 (19)	O1—C22—H22A	109.5
N1—Pd1—Cl2	88.10 (17)	O1—C22—H22B	109.5
N1—Pd1—N2	176.4 (3)	O1—C22—H22C	109.5
N2—Pd1—Cl1	89.96 (18)	H22A—C22—H22B	109.5
N2—Pd1—Cl2	90.0 (2)	H22A—C22—H22C	109.5
C22—O1—C19	118.5 (8)	H22B—C22—H22C	109.5
C41—O2—C44	117.3 (8)	N2—C23—H23	117.3
C1—N1—Pd1	124.5 (5)	N2—C23—C26	125.4 (8)
C1—N1—C2	119.6 (7)	C26—C23—H23	117.3
C2—N1—Pd1	115.9 (5)	N2—C24—H24	106.9
C23—N2—Pd1	122.7 (5)	N2—C24—C38	110.6 (6)
C23—N2—C24	115.1 (6)	C25—C24—N2	112.2 (6)
C24—N2—Pd1	121.9 (5)	C25—C24—H24	106.9
N1—C1—H1	116.7	C25—C24—C38	112.9 (6)
N1—C1—C4	126.6 (8)	C38—C24—H24	106.9
C4—C1—H1	116.7	C24—C25—H25A	109.5
N1—C2—H2	106.4	C24—C25—H25B	109.5
N1—C2—C3	109.9 (5)	C24—C25—H25C	109.5
C3—C2—H2	106.4	H25A—C25—H25B	109.5
C16—C2—N1	112.3 (6)	H25A—C25—H25C	109.5
C16—C2—H2	106.4	H25B—C25—H25C	109.5
C16—C2—C3	115.0 (6)	C27—C26—C23	119.9 (8)
C2—C3—H3A	109.5	C31—C26—C23	119.2 (8)
C2—C3—H3B	109.5	C31—C26—C27	120.7 (7)
C2—C3—H3C	109.5	C26—C27—H27	120.7
H3A—C3—H3B	109.5	C28—C27—C26	118.5 (8)
H3A—C3—H3C	109.5	C28—C27—H27	120.7
H3B—C3—H3C	109.5	C27—C28—H28	119.4
C5—C4—C1	119.5 (9)	C29—C28—C27	121.2 (8)
C5—C4—C9	120.5 (9)	C29—C28—H28	119.4
C9—C4—C1	119.7 (10)	C28—C29—H29	120.1
C4—C5—H5	119.0	C28—C29—C30	119.9 (8)
C4—C5—C6	121.9 (11)	C30—C29—H29	120.1
C6—C5—H5	119.0	C29—C30—H30	119.8
C5—C6—H6	121.4	C31—C30—C29	120.5 (9)
C5—C6—C7	117.2 (12)	C31—C30—H30	119.8
C7—C6—H6	121.4	C26—C31—C30	119.1 (10)
C6—C7—H7	118.7	C26—C31—C32	121.6 (9)
C8—C7—C6	122.5 (11)	C30—C31—C32	119.3 (10)
C8—C7—H7	118.7	C33—C32—C31	118.6 (9)
C7—C8—H8	118.4	C37—C32—C31	121.0 (9)
C7—C8—C9	123.2 (13)	C37—C32—C33	120.3 (10)
C9—C8—H8	118.4	C32—C33—H33	119.6
C8—C9—C4	114.6 (13)	C32—C33—C34	120.9 (9)
C8—C9—C10	122.1 (13)	C34—C33—H33	119.6
C10—C9—C4	123.3 (11)	C33—C34—H34	121.0
C9—C10—C11	121.9 (14)	C33—C34—C35	118.0 (10)
C15—C10—C9	123.1 (12)	C35—C34—H34	121.0
C15—C10—C11	115.0 (14)	C34—C35—H35	119.1
C10—C11—H11	119.8	C36—C35—C34	121.8 (10)
C12—C11—C10	120.3 (14)	C36—C35—H35	119.1
C12—C11—H11	119.8	C35—C36—H36	120.2
C11—C12—H12	118.7	C35—C36—C37	119.6 (10)
C11—C12—C13	122.6 (15)	C37—C36—H36	120.2
C13—C12—H12	118.7	C32—C37—C36	119.3 (9)
C12—C13—H13	120.4	C32—C37—H37	120.4
C12—C13—C14	119.2 (15)	C36—C37—H37	120.4
C14—C13—H13	120.4	C39—C38—C24	120.3 (7)
C13—C14—H14	120.0	C39—C38—C43	118.9 (8)
C15—C14—C13	120.0 (13)	C43—C38—C24	120.6 (8)
C15—C14—H14	120.0	C38—C39—H39	119.5
C10—C15—H15	118.6	C38—C39—C40	121.0 (8)
C14—C15—C10	122.7 (12)	C40—C39—H39	119.5
C14—C15—H15	118.6	C39—C40—H40	120.6
C17—C16—C2	119.0 (8)	C41—C40—C39	118.8 (8)
C17—C16—C21	115.1 (8)	C41—C40—H40	120.6
C21—C16—C2	125.9 (7)	O2—C41—C40	125.0 (9)
C16—C17—H17	118.6	O2—C41—C42	115.7 (8)
C18—C17—C16	122.8 (9)	C42—C41—C40	119.2 (9)
C18—C17—H17	118.6	C41—C42—H42	120.1
C17—C18—H18	120.4	C43—C42—C41	119.9 (8)
C19—C18—C17	119.1 (9)	C43—C42—H42	120.1
C19—C18—H18	120.4	C38—C43—H43	119.0
C18—C19—O1	114.1 (8)	C42—C43—C38	122.0 (9)
C20—C19—O1	124.8 (9)	C42—C43—H43	119.0
C20—C19—C18	121.1 (9)	O2—C44—H44A	109.5
C19—C20—H20	120.2	O2—C44—H44B	109.5
C19—C20—C21	119.6 (9)	O2—C44—H44C	109.5
C21—C20—H20	120.2	H44A—C44—H44B	109.5
C16—C21—H21	118.9	H44A—C44—H44C	109.5
C20—C21—C16	122.2 (8)	H44B—C44—H44C	109.5
			
Pd1—N1—C1—C4	−7.7 (12)	C17—C16—C21—C20	−0.6 (14)
Pd1—N1—C2—C3	105.9 (6)	C17—C18—C19—O1	179.9 (10)
Pd1—N1—C2—C16	−124.8 (6)	C17—C18—C19—C20	−0.2 (17)
Pd1—N2—C23—C26	10.4 (11)	C18—C19—C20—C21	−1.4 (16)
Pd1—N2—C24—C25	−10.7 (8)	C19—C20—C21—C16	1.8 (15)
Pd1—N2—C24—C38	116.2 (6)	C21—C16—C17—C18	−1.0 (15)
O1—C19—C20—C21	178.5 (10)	C22—O1—C19—C18	−170.9 (10)
O2—C41—C42—C43	−178.4 (9)	C22—O1—C19—C20	9.2 (16)
N1—C1—C4—C5	−46.8 (12)	C23—N2—C24—C25	175.1 (6)
N1—C1—C4—C9	139.0 (9)	C23—N2—C24—C38	−57.9 (8)
N1—C2—C16—C17	75.6 (10)	C23—C26—C27—C28	176.3 (7)
N1—C2—C16—C21	−105.0 (9)	C23—C26—C31—C30	−177.0 (8)
N2—C23—C26—C27	38.9 (11)	C23—C26—C31—C32	3.9 (13)
N2—C23—C26—C31	−146.2 (8)	C24—N2—C23—C26	−175.5 (7)
N2—C24—C38—C39	121.4 (8)	C24—C38—C39—C40	177.5 (8)
N2—C24—C38—C43	−63.2 (10)	C24—C38—C43—C42	−177.5 (8)
C1—N1—C2—C3	−76.9 (9)	C25—C24—C38—C39	−112.0 (8)
C1—N1—C2—C16	52.4 (9)	C25—C24—C38—C43	63.4 (9)
C1—C4—C5—C6	−176.8 (8)	C26—C27—C28—C29	0.4 (13)
C1—C4—C9—C8	177.2 (8)	C26—C31—C32—C33	−128.0 (9)
C1—C4—C9—C10	−4.5 (15)	C26—C31—C32—C37	52.0 (12)
C2—N1—C1—C4	175.4 (7)	C27—C26—C31—C30	−2.1 (14)
C2—C16—C17—C18	178.5 (10)	C27—C26—C31—C32	178.8 (8)
C2—C16—C21—C20	−180.0 (9)	C27—C28—C29—C30	−1.5 (14)
C3—C2—C16—C17	−157.7 (8)	C28—C29—C30—C31	0.8 (15)
C3—C2—C16—C21	21.7 (12)	C29—C30—C31—C26	1.0 (15)
C4—C5—C6—C7	1.7 (13)	C29—C30—C31—C32	−179.9 (9)
C4—C9—C10—C11	132.7 (11)	C30—C31—C32—C33	53.0 (12)
C4—C9—C10—C15	−47.4 (16)	C30—C31—C32—C37	−127.1 (10)
C5—C4—C9—C8	3.1 (14)	C31—C26—C27—C28	1.4 (12)
C5—C4—C9—C10	−178.6 (9)	C31—C32—C33—C34	179.8 (9)
C5—C6—C7—C8	−1.5 (17)	C31—C32—C37—C36	−179.3 (8)
C6—C7—C8—C9	2 (2)	C32—C33—C34—C35	−2.2 (14)
C7—C8—C9—C4	−2.9 (18)	C33—C32—C37—C36	0.6 (13)
C7—C8—C9—C10	178.8 (13)	C33—C34—C35—C36	4.1 (15)
C8—C9—C10—C11	−49.1 (16)	C34—C35—C36—C37	−3.6 (14)
C8—C9—C10—C15	130.8 (12)	C35—C36—C37—C32	1.2 (13)
C9—C4—C5—C6	−2.7 (13)	C37—C32—C33—C34	−0.1 (13)
C9—C10—C11—C12	179.9 (13)	C38—C39—C40—C41	0.9 (14)
C9—C10—C15—C14	−178.9 (10)	C39—C38—C43—C42	−2.0 (14)
C10—C11—C12—C13	−3 (2)	C39—C40—C41—O2	178.7 (9)
C11—C10—C15—C14	1.0 (15)	C39—C40—C41—C42	−3.7 (14)
C11—C12—C13—C14	5 (3)	C40—C41—C42—C43	3.7 (15)
C12—C13—C14—C15	−4 (2)	C41—C42—C43—C38	−0.9 (15)
C13—C14—C15—C10	0.9 (16)	C43—C38—C39—C40	1.9 (13)
C15—C10—C11—C12	0.0 (17)	C44—O2—C41—C40	−14.6 (14)
C16—C17—C18—C19	1.4 (18)	C44—O2—C41—C42	167.7 (8)

### (I) Hydrogen-bond geometry (Å, º)

**Table:**

*D*—H···*A*	*D*—H	H···*A*	*D*···*A*	*D*—H···*A*
C3—H3*A*···Cl1	0.96	2.90	3.662 (6)	138
C22—H22*A*···Cl1^i^	0.96	2.87	3.765 (10)	155
C25—H25*A*···Cl2	0.96	2.71	3.460 (6)	135
C44—H44*C*···Cl2^ii^	0.96	2.82	3.757 (9)	165

Symmetry codes: (i) −*x*+1, *y*−1/2, −*z*+1; (ii) −*x*, *y*+1/2, −*z*+2.
